# Sporadic multiple parathyroid gland disease—a consensus report of the European Society of Endocrine Surgeons (ESES)

**DOI:** 10.1007/s00423-015-1348-1

**Published:** 2015-11-05

**Authors:** Marcin Barczyński, Robert Bränström, Gianlorenzo Dionigi, Radu Mihai

**Affiliations:** Department of Endocrine Surgery, Third Chair of General Surgery, Jagiellonian University Medical College, 37 Prądnicka Street, 31-202 Kraków, Poland; Endocrine and Sarcoma Surgery Unit, Department of Molecular Medicine and Surgery, Karolinska Institutet, Stockholm, Sweden; First Division of Surgery, Research Center for Endocrine Surgery, University of Insubria School of Medicine, Varese, Italy; Department of Endocrine Surgery, Oxford University Hospitals NHS Trust, Oxford, UK

**Keywords:** Sporadic primary hyperparathyroidism, Multiple gland disease, Double parathyroid adenoma, Lithium-induced hyperparathyroidism, Parathyroidectomy

## Abstract

**Background:**

Sporadic multiglandular disease (MGD) has been reported in literature in 8–33 % of patients with primary hyperparathyroidism (pHPT). This paper aimed to review controversies in the pathogenesis and management of sporadic MGD.

**Methods:**

A literature search and review was made to evaluate the level of evidence concerning diagnosis and management of sporadic MGD according to criteria proposed by Sackett, with recommendation grading by Heinrich et al. and Grading of Recommendations, Assessment, Development and Evaluation (GRADE) system. Results were discussed at the 6th Workshop of the European Society of Endocrine Surgeons entitled ‘Hyperparathyroidism due to multiple gland disease: An evidence-based perspective’.

**Results:**

Literature reports no prospective randomised studies; thus, a relatively low level of evidence was achieved. Appropriate surgical therapy of sporadic MGD should consist of a bilateral approach in most patients. Unilateral neck exploration guided by preoperative imaging should be reserved for selected patients, performed by an experienced endocrine surgeon and monitored by intraoperative parathormone assay (levels of evidence III–V, grade C recommendation). There is conflicting or equally weighted levels IV–V evidence supporting that cure rates can be similar or worse for sporadic MGD than for single adenomas (no recommendation). Best outcomes can be expected if surgery is performed by an experienced parathyroid surgeon working in a high-volume centre (grade C recommendation). Levels IV–V evidence supports that recurrent/persistence pHPT occurs more frequently in patients with double adenomas hence in situations where a double adenoma has been identified, the surgeon should have a high index of suspicion during surgery and postoperatively for the possibility of a four-gland disease (grade C recommendation).

**Conclusions:**

Identifying preoperatively patients at risk for MGD remains challenging, intraoperative decisions are important for achieving acceptable cure rates and long-term follow-up is mandatory in such patients.

## Introduction

Patients with primary hyperparathyroidism (pHPT) typically have elevated serum calcium values due to excessive secretion of parathyroid hormone (PTH) from enlarged parathyroid gland(s), with inappropriate cellular regulation of the PTH secretion. pHPT is caused by a single, benign adenoma in 80–85 % of cases and by parathyroid hyperplasia or multiple adenomas (multiglandular disease) in 10–15 %, with rare occurrence of parathyroid carcinoma (<1 %). In a small group of patients (<10 %), pHPT occurs as part of a familial genetic syndrome, most commonly multiple endocrine neoplasia syndrome type 1 (MEN-1), more rarely multiple endocrine neoplasia type 2 (MEN-2) and occasionally the hyperparathyroidism-jaw tumour (HPT-JT) syndrome [1]. Although pHPT secondary to a known inherited genetic predisposition is likely to be due to synchronous or metachronous development of multiple adenomas on a background of generalised parathyroid hyperplasia, in clinical practice the majority of MGD are apparently sporadic [2]. A number of nutritional, metabolic and pharmacologic disturbances that alter parathyroid chief cell responsiveness are increasingly being recognised [2]. Some of these lead to reversible changes in the release of PTH. Some, however, appear to induce a more durable dysregulation in PTH homeostasis leading to the development of sporadic MGD [3]. This paper aimed to review controversies in the pathogenesis and management of sporadic MGD.

## Methods

A review was performed from a literature search (PubMed) concerning diagnosis and management of patients with sporadic pHPT caused by MGD. The PubMed search included articles published in the English language during recent years. Effort was made to evaluate the level of evidence to be able to depict current knowledge and new concepts of interest. Level of evidence grading was done according to criteria proposed by Sackett [4], with grading of recommendation proposed by Heinrich et al. [5], and the Grading of Recommendations, Assessment, Development and Evaluation (GRADE) system [6]. According to Sackett’s classification, the strength of a recommendation was graded ‘A’ when supported by studies with a level of evidence I (meta-analysis or large randomised trials with clear cutoff results and low risk for error); ‘B’ when supported by level II studies (small randomised trials and moderate to high risk for error); ‘C’ when supported by level III (nonrandomized, prospective with contemporaneous controls trials), level IV (non-randomised trials with historical controls, retrospective analysis) or level V studies (case series without controls, expert opinion). In the GRADE system, the strength of recommendations has been defined as ‘strong’, or ‘weak’; the quality of the evidence has been indicated by crossfilled circles: ‘⊕OOO’ denotes very low quality evidence (any estimate of effect is very uncertain); ‘⊕⊕OO’, low quality (further research is very likely to have an important impact on confidence in the estimate of effect and is likely to change the estimate); ‘⊕⊕⊕O’, moderate quality (further research is likely to have an important impact on confidence in the estimate of effect and may change the estimate); and ‘⊕⊕⊕⊕’, high quality (further research is very unlikely to change the confidence in the estimate of effect).

## Results

### Incidence of multiglandular disease in sporadic primary hyperparathyroidism

The real incidence of MGD is difficult to be defined because its estimates are influenced by several factors including the extent of parathyroid surgery (i.e. use of routine bilateral neck exploration (BNE) or limited scan-directed uni-compartmental exploration), the experience and confidence of the operating surgeon to identify MGD and the experience of the pathologist to differentiate a (micro)adenoma from a normal gland.

Historically, it was considered that up to one in five patients with pHPT might have MGD. A large retrospective review of 866 consecutive BNE operated between 1960 and 1997 reported that a single adenoma was present in 77 % of patients and hyperplasia in 21 % [7]. Similarly, in a comparative study of two American centres, MGD was reported in 16.5 % of 395 patients who underwent routine BNE and in 11 % of patients treated with focused scan-directed parathyroidectomy as the preferred strategy [8]. It is now accepted that even in patients with concordant imaging suggestive of a single adenoma, further enlarged glands could be encountered if those patients undergo formal BNE. With such a protocol applied to 350 patients, additional abnormal parathyroid glands were found on complete exploration in 15 % of patients with concordant sestamibi and ultrasound [9]. A slightly lower rate of 10 % was observed in a RCT of 46 patients, of whom 2 of 23 who had BNE were found to have an unsuspected additional enlarged contralateral parathyroid opposite to the site of the scan-localised adenoma [10]. In this context, it is not surprising that five most recent series published had a wide variable incidence of MGD ranging from 2.4 to 34 %, with variable figures over separate time periods reported even from the same centre (Table 1) [11–18].

**Table 1:** Incidence of multigland disease in recent cohorts of patients undergoing bilateral neck exploration

Reference (year)	Period, centre, operative strategy	Total number of patients	Double adenomas	Multigland hyperplasia
Alhefdhi et al. [11]	2001–2013, University of Wisconsin, USA	1402	124 (9 %)	181 MGD (13 %)
Vandenbulcke et al. [12]	1993–2010, University Hospitals Leuven, Belgium, BNE	698	46 (6.6 %)	17 (2.4 %)
Mazeh et al. [13]	2001–2010, University of Wisconsin, USA, BNE	1235	100 (8 %)	135 (11 %)
Schneider et al. [14]	University of Wisconsin, USA	1049 overt PHPT		148 (14.1 %)
		388 mild PHPT		133 (34.3 %)
Hughes et al. [15]	Ann Arbor, USA, focused parathyroidectomy with MGD discovered intraoperatively	1855		207 (11 %)
Cayo et al. [16]	2000–2008, University of Wisconsin, USA	755		163 (21.5 %)
Szabo et al. [17]	Uppsala University Hospital, Sweden, BNE	659	77 (11.7 %)	53 (8.0 %)
Attie et al. [18]	Long Island Jewish Medical Centre, USA, BNE	865	33 (3.8 %)	46 (5.3 %)

*MGD* multiglandular disease, *PHPT* primary hyperparsathyroidism, *BNE* bilateral neck exploration

These contrasting figures raise into question the clinical significance of these additional enlarged glands. If all these enlarged glands would be functionally significant, the failure rate of minimally invasive parathyroidectomy (MIP) should be much higher than the reported figures (Table 2) [19–21]. This paradox was confirmed by a comparative study between two centres undertaking routine BNE or focused parathyroidectomy, and despite their contrasting approaches, there was no statistically significant difference in their operative success: 9 of 395 (2.3 %) patients at institution A remained hypercalcemic postoperatively compared with 15 of 405 (3.7 %) at institution B (*p* = 0.24) [8].

**Table 2:** Multiglandular disease as a cause of failed minimally invasive parathyroidectomy

Ref	Centre	Total number of patients	Failure rate	Cause of failure
Lee et al. [19]	MD Anderson, USA	357	19 (3.5 %)	9 MGD
Bagul et al. [20]	Sheffield, UK	541	25 (5 %)	13 MGD (2.5 %)
Suliburk et al. [21]	University of Sydney, Australia	1020	23 (2.2 %)	10 DA, 3 MGD

*MGD* multiglandular disease, *DA* double adenoma

## Pathogenesis of sporadic multiglandular disease

### Definition of sporadic multiglandular disease

There are no reliable histologic criteria to consistently distinguish between normal, hyperplastic, and adenomatous parathyroid glands. Many authors conclude that the microscopic classification of abnormal parathyroid glands as hyperplasia or adenoma correlates poorly with the macroscopic appearance. Nevertheless, the pathologist can distinguish normal from abnormal parathyroid glands with a fair degree of accuracy. Therefore, over the years, surgeons have learned to rely on a visual gross assessment of weight, size, colour and firmness during surgery to separate the various types of pathologic involvements. A practical rule for many endocrine surgeons is that an enlarged gland is probably pathological and hypersecreting, but this may not always be true (*vide infra*).

Two discrete forms of abnormal parathyroid growth have been recognised to date; the uniglandular and the multiglandular. Uniglandular enlargement (i.e. in the presence of three remaining normal glands) represents the underlying pathology in the majority of patients, varying from 75 to 95 % of all series with pHPT. In MGD, more than one gland is involved either synchronously or asynchronously. Hyperplasia involving all four glands is the majority of pathology in MGD but two- to three-gland hyperplasia can also occur. In addition to these two entities, some consider that multiple adenomas represent a separate clinical entity but others argue that multiple adenomas do not exist and instead represent asymmetrical four-gland hyperplasia [22].

Overall, the incidence of sporadic MGD, including both multiple adenomas and hyperplasia, varies between 7 and 33 % (Table 1) [11–18].

Several attempts have been made to address the question of whether the level of PTH (or other clinical/biochemical parameter) can predict or define single-gland disease or MGD. Including variables such as serum calcium and PTH levels, results of localization studies with sestamibi and ultrasound, some studies have reported a good positive prediction value if several of these criteria are met [23], but this has not been reproduced in all studies.

Many surgeons use the observed size of a parathyroid gland as an indicator of hypersecretion. This notion is based on the excellent success and low recurrence rates achieved by excising all visually enlarged parathyroid glands during surgery.

**Fig. 1:**
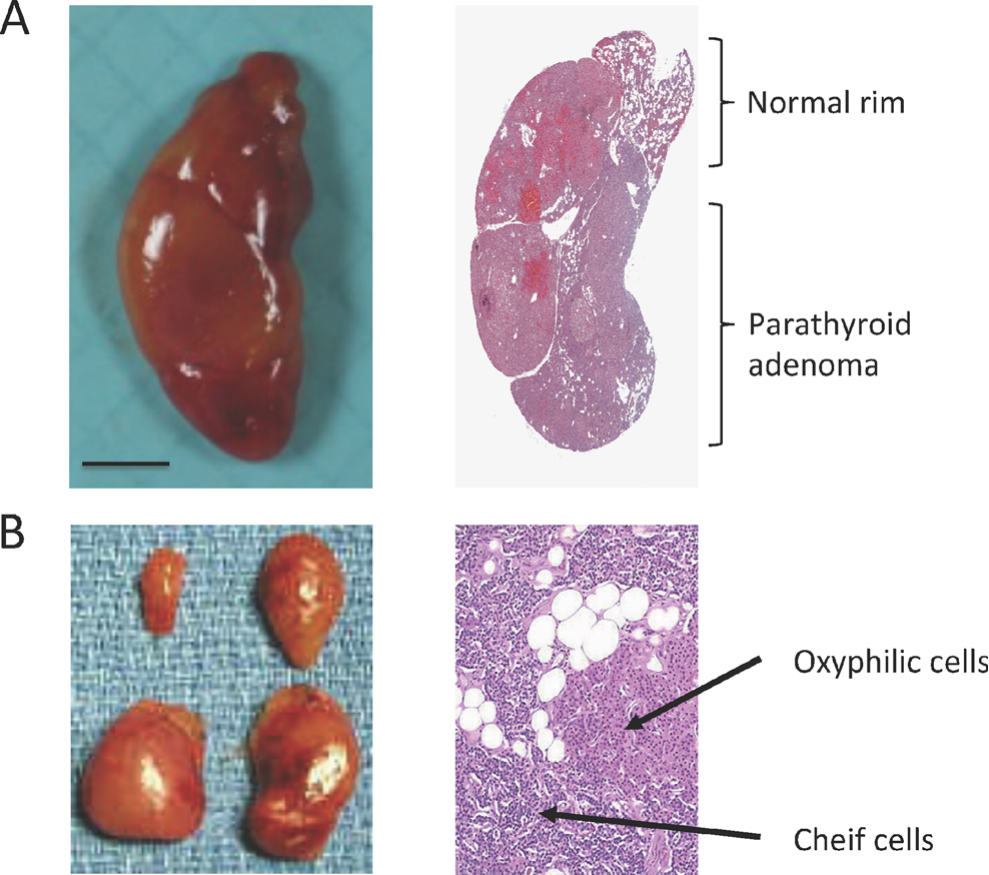
Gross macroscopic photo of a parathyroid adenoma (**a**) and four-gland hyperplasia (**b**). **a** *Right*, microscopic section of parathyroid adenoma and a normal rim. In many cases, but not all, histopathological sections in parathyroid hyperplasia show nodules containing chief and oxyphilic cells (*right* in (**b**)). Photos are shown owing to courtesy of pathologist Dr. Christofer Juhlin, Karolinska Institutet, Sweden

Many studies have tried to identify pathological criteria to distinguish between normal parathyroid tissues, adenoma and hyperplasia. Traditionally, the presence of a rim of normal parathyroid tissue adjacent to an encapsulated nodule has been the ‘gold standard’ for the diagnosis of a parathyroid adenoma (Fig. 1). However, a rim of normal parathyroid tissue is not always present and other histological characteristics have been suggested such as fibrous capsule, cellular pleomorphism, presence of nodules and mitotic figures [22, 24]. In addition, it has been shown that lipid staining may distinguish between hyperfunctioning glands from normal parathyroid tissue [25]. In normal, or suppressed, glands, chief cells exhibit abundant intracytoplasmic coarse and fine neutral lipid droplets. In hyperfunctioning tissue, droplets of intracytoplasmic neutral lipid are virtually absent [26]. Lastly, single adenomas are monoclonal lesions arising from a single precursor [27] and MGD is probably polyclonal hence they represent two different diseases.

### Double parathyroid adenomas

Double adenomas are considered to be a distinct clinical entity in-between uniglandular disease and multiglandular hyperplasia. It has been a matter of intense debate whether double adenomas represent a form of asynchronous four-gland hyperplasia. However, considering the high long-term cure rate of two-gland resection equivalent to uniglandular disease is the best evidence of double adenoma as a separate disease. A biopsy from a normal parathyroid gland is considered by many as mandatory in order to confirm the pathology present, especially in cases with multiple adenomas.

The reported incidence of double adenomas varies widely between 2 and 11 % [11–18].

Several attempts have been made to investigate if double adenoma has a different clinical pattern compared with a single adenoma and hyperplasia. Despite intensive research, no differences between patients with double adenomas and other patients with pHPT with regard to age, preoperative serum calcium and PTH levels have been established [11, 12, 28].

### Lithium-associated hyperparathyroidism

Lithium compounds are being used in long-term treatment of psychiatric diseases mainly as mood-stabilising drug and in the treatment of bipolar disorders. The mechanism of lithium-associated hyperparathyroidism (LAH) is not well understood. Many different variants of lithium salts exist, and upon ingestion, it is rapidly absorbed in the gastrointestinal channel and widely distributed in the body [29]. Lithium might directly stimulate PTH production. Alternatively, lithium presumably interferes with calcium-mediated transmembrane signal transduction by the calcium-sensing receptor, because it induces a reduction in the set point for PTH secretion. The similarity between lithium-induced hypercalcemia and familial hypocalciuric hypercalcemia (FHH), which is associated with inactivating mutations in the gene encoding the calcium-sensing receptor, has been underlined [30]. However, the exact interaction between lithium and the calcium-sensing receptor is unknown.

The prevalence of LAH varies greatly in literature from 2.7 to 23.2 % [31, 32]. The most recently published study showed a prevalence of 8.6 % for LAH [33]. The definitions used and the length of exposure to lithium can at least partly, explain the discrepancies. Though the majority of these patients (approximately 50 %) have a single parathyroid adenoma, there is a higher incidence of multiple adenomas compared with the ordinary pHPT patient cohort [33–36]. Many hypotheses on the underlying mechanism of LAH have been proposed including: increased threshold of the calcium-sensing receptor, increased secretion of the PTH, decrease of calcium uptake, inhibition of action of glycogen synthase kinase 3b and reduction of PTH gene transcription [37].

### Is sporadic multiglandular disease a synchronous or metachronous disease?

The majority of cases with multiple parathyroid adenomas are likely synchronous. This notion is based on the observation that few patients have a recurrent disease after a successful parathyroidectomy. However, detailed analysis of a large cohort of patients with pHPT by Alhefdhi and co-workers showed that the rate of persistent disease was higher among patients with double adenomas [11]. The same authors also showed that patients with double adenomas recur at a higher rate compared with patients with single adenoma and hyperplasia [11].

## Risk factors of sporadic multiglandular disease

### Age and gender

Only a few studies have specifically addressed the question of whether there are any differences in age and gender between single and double adenomas and hyperplasia. There are reports that patients with persistent or recurrent hyperparathyroidism caused by double adenoma are older and have different clinical manifestations [38], whereas other studies failed to show any differences [11, 12, 28]. However, patients with four-gland hyperplasia tended to be younger when compared with patients with parathyroid adenoma(s), but these differences were not significant. To summarise, present data do not support any differences with respect to gender, age and preoperative serum calcium and PTH levels between uniglandular disease and MGD.

### Radiation

Several cohort studies have shown that radiation towards the neck area increases the risk for pHPT [39, 40], whereas others have failed to do so [41]. Recently, Boehm and Dietrich showed that up to 25 % of 61 liquidators, or cleanup workers, had signs of hyperparathyroidism 14 years after the nuclear plant accident in Chernobyl [42]. The risk of pHPT associated with radiation exposure in this cohort of liquidators was significantly higher (*p* < 0.001) when compared with the overall prevalence of pHPT in a non-exposed background population (as reported for incidence in the US population in 2001), with an odds ratio of 63.4 (95 % CI, 35.7–112.5) [42]. However, these studies do not report the outcome after parathyroidectomy, and there is no information whether radiation-induced hyperparathyroidism has an increased prevalence of MGD. Tezelman and co-workers reported no differences in hyperplasia or double adenoma between sporadic and radiation-induced hyperparathyroidism [43]. Taken together, it is fair to conclude that ionising radiation is capable of inducing parathyroid neoplasms, but the discrepancy between studies most likely reflects the diversity of radiation type, targeted organs, doses and the observation interval.

### Are there any other known risk factors for sporadic multiglandular disease?

There are a number of known risk factors for hyperparathyroidism, like chronic renal failure, vitamin D deficiency, lithium medication and less commonly chronic pancreatitis, small bowel disease and malabsorption-dependent bariatric surgery. These diseases are classified as secondary HPT to denote a known cause of hyperparathyroidism.

As discussed above, several studies failed to identify any correlation between MGD and symptoms, age, serum calcium and PTH levels, nor clinical presentation. However, it is tentative to speculate that pHPT with multiglandular involvement is caused by an underlying signal yet to be elucidated, especially for four-gland hyperplasia. But, also for multiple adenomas, it seems unlikely that two neoplasms occurs spontaneously. Considering the risk of a pHPT in a cohort of patients is 2–3 %. By pure chance, the risk of developing two adenomas in the same cohort should be less than 0.1 %, i.e. several fold lower than the observed incidence hence their occurrence is not due to chance.

## Preoperative diagnosis of sporadic multiglandular disease

### Can sporadic multiglandular disease be diagnosed preoperatively?

Parathyroid glands can be imaged with multiple modalities, including scintigraphy, high-resolution ultrasonography (US), thin-section computed tomography (CT) and magnetic resonance imaging (MRI) [44]. US and parathyroid scintigraphy with methoxyisobutylisonitrile (sestamibi) are the dominant imaging techniques used in the setting of pHPT. Sestamibi (with pinhole collimator plus early/late acquisition) is the recommended first test, but US by an experienced investigator (radiologist, endocrinologist, surgeon) is an alternative [45]. The second test (sestamibi or US) is used to confirm the result of the first investigation. CT and MRI are generally useful additional imaging modalities in the case of ectopic mediastinal parathyroid adenomas since they provide detailed anatomical localization of ectopic mediastinal lesions for surgical planning [46].

Evaluation of patients with combined modalities is gaining clinical importance [45]. Combined interpretation of scintigraphy and US, or scintigraphy and CT, can improve the diagnostic interpretation of parathyroid scintigraphy and clinical decision making [47]. Several investigators confirmed that sporadic MGD cannot be diagnosed preoperatively due to low accuracy, sensitivity and specificity of any preoperative localisation tests performed [48–62]. In particular, routine sestamibi scintigraphy and US alone or combined do not reliably predict MGD [44, 46–49, 52, 63].

Negative preoperative localization studies are highly predictive of MGD. A study investigated whether negative localization studies select a specific population of patients [23]. Patients with negative preoperative study results had a high risk of MGD (31.6 %) compared with patients with one positive study result (3.6 %) and those with two concordant positive study results (0.0 %; *p* < 0.001) [63]. Moreover, if the diagnosis of pHPT remains unequivocal the persistent or recurrent disease is more likely due to parathyroid hyperplasia than solitary adenoma [45].

Several authors have explored the possibility of predicting pre- or intraoperatively the presence of MGD based on scoring models.

Kebebew et al. at San Francisco described a dichotomous scoring model based on preoperative total calcium level (≥3 mmol/L (≥12 mg/dL)), intact parathyroid hormone level (≥2 times the upper limit of normal levels), positive ultrasound and sestamibi scan results for one enlarged gland, and concordant ultrasound and sestamibi scan findings. The model was derived from data collected on 238 patients, of whom 75.2 % had a single adenoma, 21.4 % had asymmetric four-gland hyperplasia and 3.4 % had double adenomas [23]. The positive predictive value of this scoring model to correctly predict single-gland disease was 100 % for a total score of three or higher. The authors suggested that patients with a score of three or higher can undergo a minimally invasive parathyroidectomy without the routine use of intraoperative parathyroid hormone or additional imaging studies, and those with a score of less than 3 should have additional testing to ensure that multiglandular disease is not overlooked [62]. The usefulness of the Kebebew scoring model was validated by Elarai et al. at the same institution in a twofold larger cohort of patients (*n* = 487) [64], and independently by Kavanagh et al. in a cohort of 180 patients treated in Ireland [65].

Preoperative oral calcium-loading test was proposed as an adjunct in the differential diagnosis between adenoma and hyperplasia. After oral administration of 1 g of calcium gluconolactate, 32 patients and 32 controls had calcium and PTH measured before and at 60, 120 and 180 min afterwards. PTH decline <30 %, product *P* (minimal PTH concentration (pg/mL) × maximal calcium concentration (mg/dL)) > 1100, and ratio *R* (relative PTH decline/relative calcium increase) < 4 diagnosed adenoma with specificity of 100, 90 and 100 %, respectively. PTH decline >60 % diagnosed hyperplasia with specificity of 100 %. The total accuracy of the test was 65 % [66]. This model has not been confirmed by other groups.

Chen et al. at Wiscosin University proposed the Wisconsin Index (WIN), defined as the multiplication of preoperative serum calcium by preoperative PTH. Patients were divided into three WIN categories: low (<800), medium (801–1600) and high (>1600). Data from 1235 patients was used to derive a WIN nomogram, consisting of the combination of WIN and parathyroid gland weight. This nomogram accurately predicted the likelihood of additional hyperfunctioning parathyroid glands. For example, for a WIN of less than 800 and a gland weight of 500 mg, there is a 9 % chance for additional hyperfunctioning parathyroid glands based on the WIN nomogram. In contrast, for the same gland weight, if the WIN is 801 to 1600, these chances increase to 28 %, and if the WIN is more than 1600, the chance of multiglandular disease is 61 %. This simple intraoperative tool may be used to guide the decision of whether to wait for intraoperative PTH assay (IOPTH) results or to proceed with further neck exploration [13].

Most recent development was proposed by Udelsman et al. from Yale University [67]. A mathematical model for pHPT was developed and embedded in a software to yield intraoperative predictability curves. A cohort of 617 patients (554 single adenoma (SA) and 63 MGD) was used to generate an idealised model that was embedded in software and installed in a laptop computer to enable intraoperative decision analyses, PTH curve plotting, and storage and transmission of data. A subsequent cohort of 100 consecutive unselected patients (81 single adenomas and 19 MGD including 13 cases of hyperplasia, two MEN-1, one lithium, three double adenomas) were tested using this model. The model predicted an overall curative resection in 95 % of patients. In single adenoma patients, cure was predicted in 78/81 patients with a mean probability of 99.3 % at 11.8 ± 10.4 min postresection. The model also correctly predicted residual hyperfunctioning tissue in all tested multiglandular patients. All MGD patients underwent additional exploration with resection of residual disease resulting in a mean predicted cure rate of 97.9 % at 10.6 ± 7.3 min postresection completion in 17 patients. This intraoperative prediction software expedites termination of surgery with a high level of curative confidence. Alternatively, the model accurately predicts residual disease prompting additional exploration. Because the model is based on a large set of multivariate regression curves, PTH values obtained at any postresection sampling interval generate prediction data with far greater accuracy than existing algorithms. The software is designed for convenient operative use and can print, store and electronically transmit probability analyses and PTH curves in real-time [67].

### Can sestamibi distinguish single-gland from sporadic multiglandular disease?

Sestamibi scan has a high sensitivity to localise a solitary parathyroid adenoma in patients even with mild increase in serum calcium level [68]. The sensitivity decreases significantly in patients with MGD and concomitant thyroid nodular abnormalities [69]. In one study, sestamibi subtraction scintigraphy correctly localised 31/36 (86 %) parathyroid adenomas compared with only 17/36 (47 %) by thallium subtraction scintigraphy (*p* < 0.001) [68]. There was one false-positive result in the sestamibi group because of a thyroid adenoma, and two of the scans were negative. Both the sestamibi and the thallium subtraction scintigraphy localised one single enlarged gland in all three patients with multiple gland involvement. In no case was MGD predicted.

In another study form Bergenfelz et al. [70] in the six patients with incorrect scans, two patients with solitary parathyroid adenoma were not correctly lateralized and four patients had asymmetric hyperplasia with two enlarged glands each [70]. Thus, in no patient was MGD predicted by the scan.

The reason for this low sensitivity is not clear and wide range of accuracy for sestamibi scintigraphy has been reported in a meta-analysis [71]. The sensitivity of sestamibi scintigraphy has been associated with the size of the parathyroid adenoma, the level of serum calcium and PTH, as well as the oxyfilic content of tumour, and concomitant thyroid disease [72]. The data from Nichols et al. showed MIBI scintigraphy to be significantly less sensitive (61 vs. 97 %) and less specific (84 vs. 93 %) for detecting MGD than single-gland disease and that the sensitivity of the test decreases progressively as the number of the lesion increased (Tables 3 and 4) [73, 74]. The possible factor in explaining the reduced MIBI sensitivity and specificity for MGD could be: (a) weight of parathyroid lesion: parathyroid lesions in multiple gland disease are smaller than in singular gland disease; (b) histology: MGD is usually due to hyperplasia whereas singular gland disease is due to adenoma and MIBI imaging has been reported to be less sensitive for detecting hyperplastic than adenomatous glands [74–77].

**Table 3:** Scintigraphy is significantly less sensitive and less specific for detecting MGD than singular gland disease

Prelesion test characteristics for all patients			
	MGD lesions	SGD lesions	All lesion
Sensitivity	61 %* (201/331)	97 % (503/520)	83 % (704/851)
Specificity	84 %* (163/193)	93 % (1450/1560)	92 % (1613/1753)
Accuracy	69 %* (364/524)	94 % (1953/2080)	89 % (2317/2604)
Positive predictive value	87 % (201/231)	82 % (505/613)	83 % (704/844)
Negative predictive value	56 %* (163/293)	99 % (1450/1467)	92 % (1613/1760)

Modified form refs. [73, 74]

*MGD* multiglandular disease, *SGD* single gland disease

*p < 0.05 MGD vs. SGD

**Table 4:** The sensitivity of the test decreases progressively as the number of the lesion increases

Effect of increasing lesion number per patient on test performance				
	1 lesion	2 lesions	3 lesions	4 lesions
Sensitivity	97 % (503/520)	68 %* (100/148)	59 %* (79/135)	46 %* (22/48)
Specificity	93 % (1450/1560)	84 %* (124/148)	87 %* (39/45)	
Accuracy	94 % (1953/2080)	76 %* (224/296)	66 %* (118/180)	46 %* (22/48)
Positive predictive value	82 % (505/613)	81 % (100/124)	93 %* (79/85)	100 % (22/22)
Negative predictive value	99 % (1450/1467)	72 %* (124/172)	41 %* (39/95)	0 %* (0/26)

Modified form refs. [73, 74]

*p < 0,05 vs. one lesion

### What is the risk of sporadic multiglandular disease when a sestamibi scan is negative?

Most published reports regarding this issue are contemporaneous or historical controls, retrospective studies, single institutional series or cohort studies with less than 50 cases. In some reports, the small number of cases and events precluded the application of statistical analysis. Some data that would be informative, such as symptoms at presentation, medical history, patient comorbidities, radiographic studies, intent of surgery, operative reports, rates of recurrence and pertinent serologic laboratory values, were not analysed because they were not collected in the database.

There is only one large series with extensive data [72]. Results from the preoperative investigations with sestamibi scintigraphy revealed: MGD was not predicted in 55/1474 patients (3.7 %); while there was a false prediction of MGD in solitary adenoma in 6/1473 patients (0.4 %) and a correct prediction of MGD in 10/1473 patients (0.7 %). Negative examinations were 351/1473 (23.0 %) [72]. In comparison, results from the preoperative investigations with ultrasound revealed: correct position of one pathologic gland, but MGD not predicted in 27/1120 patients (2.4 %); false prediction of MGD in solitary adenoma in 7/1120 patients (0.6 %); and correct prediction of MGD in 10/1120 Patients (0.9 %). Negative examinations were 348/1120 (31.0 %) [72]. Therefore, if investigation with sestamibi scintigraphy and ultrasound is negative, surgery is probably more difficult than for patients with no localization investigation, or with a positive localization test.

Negative localization with sestamibi and ultrasound in pHPT infers a highly selected patient population with small parathyroid adenomas, an alarmingly high rate of negative exploration and an increased risk for persistent disease with outcome inferior than standards. This was confirmed in a study of 213 patients operated for pHPT after dual scanning with sestamibi and US [62]. When at least one study showed a positive result (*n* = 175), the patient underwent a video-assisted approach with IOPTH monitoring. When results were negative (*n* = 38), the patient underwent cervicotomy and exploratory procedures of all four parathyroid glands. All patients were cured (mean follow-up, 17.8 ± 10.3 months). Patients with negative preoperative study results had a high risk of MGD (12/38 patients; 31.6 %), compared with patients with one positive study result (3/83 patients; 3.6 %; *p* < 0.001) and those with two concordant positive study results (0/92 patients; *p* < 0.001). The authors concluded that when preoperative localization study results are negative, the patient has a high risk of MGD, and a conventional cervicotomy with identification of the four glands is recommended strongly. When only one localization study is positive, the risk of MGD justifies the use of IOPTH monitoring during a focused approach. When positive localization study results are concordant, the use of IOPTH is questionable during a focused approach [62].

### How accurate is the use concordant sestamibi scan and ultrasound for distinguishing single gland from sporadic multiglandular disease?

Combined sestamibi and US can increase the accuracy of localization of a single adenoma from 94 to 99 %. When concordant, sestamibi and US localization has been reported to have an operative success rate approaching 99 % [78–81]. Discordance between sestamibi and US has been reported to be as high as 38 % in consecutive patients treated by parathyroidectomy, with an 11 % rate of MGD [82]. Although the sensitivities for both localising studies for MGD are lower, the risk of missing abnormal glands can be minimised by utilising IOPTH monitoring [82].

### What is the accuracy of CT scan for distinguishing single-gland disease from sporadic multiglandular disease?

CT is advised when an ectopic, mediastinal parathyroid lesion is seen on sestamibi, in previous neck surgery and/or coexisting thyroid disease [77, 83–85]. Most studies have investigated its ability to localise adenomas rather than to identify MGD.

More recently, 4D-CT scan has been described to be a very sensitive technique [86]. The name is derived from 3D-CT with an added dimension from the changes in perfusion of contrast over time. 4D-CT utilises multiplanar images and perfusion characteristics to identify abnormal parathyroid glands [86]. By evaluating for an early enhancing and early washout of parathyroid glands, individual enhancement characteristic can then be correlated with metabolic activity, which allows 4D-CT to demonstrate gland functionality in addition to providing anatomic details.

Few studies assessed the role of 4D-CT in patients with inconclusive preoperative ultrasound and sestamibi localization studies. The study from Philip et al. demonstrated that 4D-CT scan had improved sensitivity (88 %) over sestamibi imaging (65 %) and ultrasonography (57 %) in localising hyperfunctioning parathyroid glands [82]. Table 4 shows the values of sensitivity and specificity that emerged from the study of Rodgers et al. in which 11 of 75 patients had MGD. 4D-CT identified ≥2 glands in five of these patients (45 %). This was in contrast to sestamibi, which identified ≥2 glands in only one patient [77]. In the study of Lubitz et al., comprising 60 patients, 4D-CT accurately lateralized 73 % and localised 60 % of abnormal glands found at operation (Table 5). Single candidate lesions (46/60) were confirmed at operation in 70 %. When multiple lesions were identified on 4D-CT (14/60), accuracy dropped to 29 % (*p* = 0.03). The accuracy of 4D-CT was not different between primary and reoperative cases (*p* = 0.79). Of the eight patients with MGD diagnosed perioperatively, five had multiple candidate lesions noted on 4D-CT. In 94 % (48/51) of patients, a >50 % drop in IOPTH level was achieved after resection and 87 % (48/55) had long-term cure with a median follow-up of 221 days. Authors concluded that 4D-CT identifies more than half of abnormal parathyroids missed by traditional imaging and should be considered in cases with negative or discordant sestamibi and ultrasound [86]. Bilateral exploration is warranted when multiple candidate lesions are reported on 4D-CT. However, taking into consideration that 4D-CT carries a huge radiation exposure, BNE remains the gold standard surgical approach having a high success rate in experienced hands in cases with negative or discordant sestamibi and ultrasound (grade C recommendation, GRADE: high, ‘⊕⊕⊕⊕’).

**Table 5:** Values of sensitivity and specificity that emerged with 4D-CT scan

Sensitivity and specificity of imaging for localization of parathyroid tumours to side of the neck and quadrant of the neck				
Variable	Sensitivity (95 %)	95 % CI	Specificity (%)	95 % CI
Side of the neck				
4D-CT	88	81–95	88	80–96
Ultrasonography	57	47–67	94	88–99
Sestamibi	65	55–75	88	80–96
Precise location in the neck				
4D-CT	70	59–81	89	85–93
Ultrasonography	29	20–38	86	82–90
Sestamibi	33	24–42	83	79–87

Modified from ref. [77]

### How accurate is SPECT imaging for distinguishing single gland from sporadic multiglandular disease?

SPECT/CT provides fused images of functional and anatomical modalities which considerably improve the interpretation of findings of individual procedures [87–89]. This innovation might improve the relatively poor results obtained in the detection of multiglandular hyperplastic disease, but further data are needed to establish its role in the field. In 2009, Wimmer et al. analysed the sensitivity and specificity of CT-MIBI-SPECT in 30 patients [75]. The aim of this study was to evaluate whether CT-MIBI-SPECT image fusion is superior to MIBI-SPECT alone and CT alone in detecting abnormal parathyroid tissue in patients with MGD (Table 6). There were six patients with pHPT (4 MEN-1 syndromes and 2 double adenomas; 1 of these patients had HRPT2 gene mutation), 14 with secondary, 8 with tertiary HPT and 1 patient each suffering from persistent primary and persistent secondary hyperparathyroidism. In five out of six patients with MGD, more than one gland was detected, thus MGD could be suspected preoperatively. Overall, CT-MIBI-SPECT image fusion was able to predict the exact position of all abnormal glands per patient in 14 of 30 (46.7 %) cases, whereas CT alone was successful in 11 (36.7 %), and MIBI-SPECT alone just in four (13.3 %) of 30 patients. This study demonstrated that CT-MIBI-SPECT image fusion is superior to CT or MIBI-SPECT alone in preoperative localization of all pathologic glands in patients suffering from MGD [75].

**Table 6:** Accuracy of SPECT imaging for distinguishing single gland from sporadic MGD

SGD vs. MGD							
Statistic and disease	Early images	Late images	Subtract on images	SPECT images	Early and late images	Planar images	All images
Sensitivity (%)							
SGD	74 (303/400)*	87 (355/409)*	88 (360/409)*	90 (369/409)*	90 (370/409)*	96 (392/409)*	90 (369/409)*
MGD	63 (79/125)	65 (81/125)	54 (68/125)	59 (74/125)	61 (76/125)	63 (79/125)	66 (82/125)
Specificity (%)							
SGD	93 (433/464)	92 (427/464)	94 (435/464)	84 (390/464)	90 (419/464)	89 (410/464)	98 (453/464)*
MGD	82 (9/11)	82 (9/11)	73 (8/11)	82 (9/11)	82 (9/11)	64 (7/11)	73 (8/11)
Accuracy (%)							
SGD	84 (736/873)*	90 (782/873)*	91 (795/873)*	87 (759/873)*	90 (789/873)*	92 (802/873)*	94 (822/873)
MGD	65 (88/136)	66 (90/136)	56 (76/136)	61 (83/136)	63 (85/136)	63 (86/136)	66 (90/136)

Modified form ref. [75]

*MGD* multiglandular disease, *SGD* single gland disease

*p < 0.05 for comparison with data for MGD

### Is genetic testing justified in patients with seemingly sporadic multiglandular disease under 40 years of age to rule out hereditary parathyroid disease?

MEN-1 is an autosomal dominant disorder characterised by the occurrence of tumours of the parathyroid, enteropancreatic neuroendocrine tissues and anterior pituitary. It is a rare disease with an estimated prevalence of 0.01–2.5 cases per 1000 individuals [90]. The presentation of MEN-1 occurs within the context of previously identified kindred, in a newly ascertained individual with advanced disease who might be the proband of new kindred or as a de novo mutation. Comparing tumours in the same tissues, they usually appear one to two decades earlier in the familiar forms compared with the sporadic ones [91]. The *MEN-1* gene was identified in 1997 and consists of 10 exons on chromosome 11q13 encoding a 610-amino acid protein known as Menin. More than 1300 mutations have been identified in the *MEN-1* gene to date, and there is no evidence of genotype-phenotype correlations (as in MEN-2) [92, 93].

The prevalence of MEN-1 among patients with apparently sporadic component tumours varies widely by tumour type. Approximately 1/3 of patients with Zollinger-Ellison syndrome will carry a MEN-1 mutation [90]. In individuals with apparently isolated hyperparathyroidism or pituitary adenomas, the mutation prevalence is lower (2 % to 5 %), but the prevalence is higher in individuals diagnosed with these tumours at younger ages (<40 years old) [94–96]. Some authors suggest MEM-1 testing in those not meeting diagnostic criteria if one of the following is present: gastrinoma at any age, multifocal pancreatic islet cell tumours at any age, parathyroid adenomas before age of 40 years, multiglandular parathyroid adenomas or recurrent hyperparathyroidism or individuals with one of the three main MEN-1 plus one of the less common findings [92, 96]. Balogh K et al. summarised the indications for genetic screening for MEN-1 (Table 7) [76]. The methods of screening are outside the remit of this paper.

**Table 7:** Summary of the indications for genetic screening

Clinical manifestation of MEN-1 syndrome and indications for genetic screening		
Major lesion (prevalence)	Minor lesions	Indications for genetic screening
Hyperparathyroidism (90–97 %)	Adrenal adenomas	*Index case*
Pituitary adenoma (33 %)	Facials angiofibromas	Clinically defined MEN-1
Tumours of endocrine pancreas (30–80 %)	Lipomas	(2 major lesions; 3 major and minor lesions)
	Neuroendocrine carcinoids	Clinically suspicious or atypical MEN-1
	Thyroid neoplasms	
	Phaeochromocytomas	
	Malignant melanomas	*Member of a MEN-1 family*
	Testicular teratomas	All first degree relatives: a relative who shows signs or symptoms of MEN-1

Modified from ref. [76]

## Surgical treatment of sporadic multiglandular disease

### Bilateral neck exploration for sporadic multiglandular disease

Although most patients with pHPT are ideal candidates for MIP, some will have more than one enlarged gland and require BNE to achieve biochemical cure. Interestingly, the evidence is unclear as to whether all enlarged parathyroid glands are hyperfunctioning, because no prospective study has been done without removing such glands to determine if patients are at risk for persistent or recurrent disease [3].

In a systematic review including 2166 patients from 14 studies who underwent BNE, 79.7 % had a single adenoma and 19.3 % had multiglandular disease. Of 2095 patients in 31 studies with a focal unilateral approach, 92.5 % had a single adenoma, whereas only 5.3 % had MGD. Thus, the incidence of MGD was significantly lower among patients treated with a focal unilateral approach compared with a bilateral approach (*p* < 0.001). Hence, it was postulated that a focal unilateral surgical approach for pHPT might underestimate the incidence of MGD [97]. Actually, more recent data do not confirm an increased prevalence of recurrent hyperparathyroidism among patients initially treated by focal approach. Schneider et al. analysed retrospectively 1368 parathyroid operations for pHPT including 1006 MIPs and 380 BNEs. There were no differences in recurrence between MIP and BNE groups (2.5 vs. 2.1 %; *p* = 0.68), and the operative approach did not independently predict recurrent disease in the multivariable analysis [98]. Hence, it should be taken into consideration that some grossly enlarged and histologically abnormal parathyroid glands can be non-functional and BNE may lead to overtreatment at least in some patients with pHPT.

There is no level I or II evidence to answer the question of which patients with pHPT should undergo BNE. However, it should be considered in MEN-1 syndrome, negative preoperative localization studies and inadequate decrease of IOPTH level following removal of the image-indexed parathyroid lesion. All other clinical scenarios can be regarded as relative indications for BNE and include isolated familial PHPT, MEN-2 syndrome, history of lithium therapy, history of head and neck irradiation or discordant preoperative localization studies (grade C recommendation, GRADE: moderate, ⊕⊕⊕O).

### Is there a place for minimally invasive surgery in lithium-associated hyperparathyroidism?

The overall incidence of MGD calculated from several recent series of LAH was 51 %, ranging from 4/16 (25 %) [99], 6/19 (32 %) [100], 36/71 (52 %) [34], 27/48 (56 %) [101] and 16/27 (62 %) [102] (Table 8). For this reason, many advocate BNE for all such patients, although the use of IOPTH might allow for a more limited exploration in selected patients who demonstrate an appropriate intraoperative fall in PTH.

**Table 8:** Surgical management of LAH based on recent publications

Reference (year)	No of patients with LAH	IOPTH	Pathology (%)			Surgery (%)		p/rHPT (%)	Perm. HypoPT (%)
			SA	DA	PH	BNE	Scan-directed		
Wade et al. [100]	19	Yes	68	32 (MGD)		53	47	100	0
Marti et al. [102]	27	Yes	41	15	44	67	33	23	0
Skandarajah et al. [101]	15	Yes	27	0	73	80	20	0	7
Järhult et al. [34]	71	N/A	45	3	52	97	3	42	13
Carchman et al. [99]	16	Yes	75	12.5	12.5	50	50	0	0

*LAH* lithium-associated hyperparathyroidism, *SA* single adenoma, *DA* double adenoma, *PH* parathyroid hyperplasia, *MGD* multiglandular disease, *BNE* bilateral neck exploration, *p/rHPT* permanent/recurrent hyperparathyroidism, *Perm.HypoPT* permanent hypoparathyroidism

In a large cohort of 1207 consecutive patients, the rate of MGD was not higher in LAH: present in 25 % (4/16) patients with LAH and 12.3 % (146/1191) patients without LAH (*p* = 0.13). Among 16 patients with LAH, 12 (75 %) had a single adenoma. The use of IOPTH allowed unilateral exploration in 8 of 12 patients with single adenoma. Parathyroid exploration resulted in durable biochemical cure for all 16 patients with LAH. Authors concluded that MGD seems to be no more frequent in patients with LAH than in patients with pHPT without LAH, and patients with LAH can be safely and effectively managed with selective unilateral exploration directed by intraoperative IOPTH [99].

However, the eligibility of LAH patients for unilateral neck exploration is still a matter of debate. Marti et al. presented retrospective analysis of 27 patients with LAH undergoing parathyroidectomy with the IOPTH. Cervical exploration was unilateral in 9, bilateral in 18 (three were converted from unilateral). Twenty-five (92.6 %) of 27 patients had initially successful surgery. Of the 17 patients with >6 months follow-up, two had persistent disease (11.8 %) and two (11.8 %) experienced recurrent disease. All patients with a single adenoma remain free of disease. Three (75 %) of four patients with persistent/recurrent disease had multiglandular disease and were receiving lithium at the time of surgery. Patients with persistent/recurrent disease were older (*p* = 0.01) and had experienced a longer duration of hypercalcemia (*p* = 0.04). Based on their outcomes authors concluded that LAH patients have a high incidence of MGD, and bilateral exploration is frequently necessary. With access to the IOPTH, it is reasonable to initiate a unilateral approach because many patients will harbour single adenomas and can be reliably rendered normocalcemic. Patients with MGD remain at higher risk of persistent/recurrent disease [102].

Wade et al. performed a retrospective review of a prospective, single institution parathyroid database of 1010 patients who underwent parathyroidectomy between December 1999 and October 2010. Nineteen (1.9 %) patients with a history of lithium therapy and sporadic pHPT were identified. A total of 18 patients underwent preoperative imaging. Of 12 (67 %) patients with single-site localization, 6 (50 %) underwent a minimally invasive parathyroidectomy, 2 (17 %) underwent unilateral explorations, 1 (8 %) underwent bilateral exploration and 3 (25 %) had concomitant thyroidectomies. Six patients did not localise and underwent bilateral exploration for multiglandular disease. One patient without preoperative imaging had single-gland disease. In all operations, surgeons used IOPTH and met intraoperative criteria. Median IOPTH decrease was 74 % (54–86) in single-gland disease and 85 % (76–95) in MGD. Median abnormal gland weight was 590 mg (134–6750 mg) in single-gland disease and 296 mg (145–2170 mg) in MGD. All patients were normocalcemic at a median follow-up of 19 months (2–118). Authors concluded that of 19 patients with lithium exposure, 6 (32 %) had MGD. However, of the 13 (68 %) patients with a single-gland disease, all 12 who had preoperative imaging had single-site localization. Thus, if localization suggests single-gland disease, minimal invasive parathyroidectomy with IOPTH can be successfully performed [100].

More limited confidence in MIP for LAH was presented by Skandarajach et al. who reviewed their multi-institutional experience based on surgical treatment of 15 patients with LAH. All 15 patients had preoperative imaging: sestamibi scanning showed that 10 (67 %) patients had localised single-gland disease, 1 (7 %) had multiple hot spots and 4 (27 %) had a negative scan. Ultrasonography demonstrated a single abnormal gland in 8 (50 %) patients and multiple enlarged glands in 1 (7 %) patient; the test was negative in 6 (40 %). As a consequence of concordant preoperative imaging a minimally invasive approach (endoscopic or a focused lateral approach) was adopted in 3 (20 %) patients. Focused surgery demonstrated an enlarged hyperplastic gland in all three cases and resulted in normocalcemia in the immediate postoperative period. None of these patients showed evidence of recurrence at follow-up. Thus, LAH is predominantly a MGD characterised by asymmetrical hyperplasia that is frequently associated with misleading or discordant localization studies. BNE is therefore recommended in order to minimise the risk of disease recurrence [101].

Järhult et al. analyzed retrospectively the long-term outcome after surgery for LAH in a large series of patients. Seventy-one patients on chronic lithium therapy who underwent surgery in three university and three district hospitals in Sweden were followed for a median of 6.3 years. The primary histopathological diagnoses were adenoma (45 %), double adenomas (3 %) and hyperplasia (52 %). Thirteen per cent of the patients suffered from permanent hypoparathyroidism. At follow-up, the rate of persistent and recurrent HPT was 42 % regardless of the histopathological diagnosis. The authors concluded that the results of conventional surgery for LAH are poor. The surgical approach should be adjusted for the MGD that is usually the cause of HPT in patients on chronic lithium therapy [34].

Thus, there is conflicting and equally weighted low level evidence supporting a routine preoperative plan of bilateral neck exploration vs. selective unilateral exploration for LHA (no recommendation) [3].

### Is IOPTH monitoring helpful for the detection and postoperative outcome prediction in sporadic multiglandular disease?

The IOPTH assay is widely utilised to confirm complete removal of all hyperfunctioning parathyroid tissue, which allows for termination of surgery with confidence that the hyperparathyroid state has been successfully corrected and to identify patients with additional hyperfunctioning parathyroid tissue following the incomplete removal of diseased parathyroid/s, hence minimising the risk of operative failure.

Understanding the nuances of IOPTH monitoring allows surgeons to achieve intraoperative confidence in predicting operative success and preventing failure in cases of unsuspected MGD, while safely limiting neck exploration in the majority of patients with sporadic pHPT. When concordant results of functional imaging (e.g. sestamibi scanning) and ultrasound performed by an experienced investigator are obtained, MIP can be safely recommended [103, 104]. The prevalence of MGD among patients with pHPT and concordant imaging tests varies from 1 to 3.5 % [104, 105]. Thus, when preoperative localization with sestamibi and ultrasound is concordant for single-gland disease, the use of IOPTH monitoring is of little value. However, if preoperative localization with sestamibi and ultrasound is not concordant and the surgeon wishes to perform a minimally invasive ‘selective’ operation, the use of IOPTH monitoring is recommended, as the prevalence of MGD in this subgroup of patients with pHPT approaches 17 % [109–112]. Similarly, the use of IOPTH monitoring is recommended for patients undergoing selective parathyroidectomy on the basis of a single preoperative localization study [106].

On the other hand, the accuracy of IOPTH monitoring in the detection of patients with MGD is highly dependent on the criteria applied. A retrospective validation of different IOPTH criteria have shown that the Miami criterion followed by the Vienna criterion is the best balanced among other criteria, with the highest accuracy in intraoperative prediction of cure [104]. However, the Rome criterion followed by the Halle criterion is most useful in intraoperative detection of multiglandular disease [104, 107–109] (Table 9). Nevertheless, their application in patients qualified for minimally invasive parathyroidectomy with concordant results of sestamibi scanning and ultrasound of the neck would result in a significantly higher number of negative conversions to bilateral neck explorations and only a marginal improvement in the success rate of primary operations [104]. Thus, the accuracy of IOPTH monitoring is highly dependent on the criteria used by the surgeon to predict the outcome of parathyroid surgery. In addition, Carneiro-Pla et al. underlined that histopathology of excised abnormal parathyroid glands did not predict the secretory function of the remaining parathyroid glands left in situ. IOPTH guided parathyroidectomy accurately based on function alone; however, histopathology was inaccurate in predicting MGD and should not be used to guide parathyroidectomy in patients with sporadic pHPT [107].

**Table 9:** IOPTH predictive values when using different criteria

Reference (year)	MGD/SA (%)	Criterion	PPV (%)	NPV (%)	Conclusion
Barczynski et al. [104]	9/260 (3.5)	Halle	100	14.2	Miami criterion followed by the Vienna criterion is the best balanced among other criteria, with the highest accuracy in intraoperative prediction of cure. However, the Rome criterion followed by the Halle criterion is most useful in intraoperative detection of MGD.
		Miami	99.6	70	
		Rome	100	26.3	
		Vienna	99.6	60.9	

*MGD* multiglandular disease, *SA* single adenoma, *PPV* positive predictive value, *NPV* negative predictive value

Nevertheless, controversy remains over the utility of IOPTH in MGD, with concern for false-positive results leading to prematurely concluding the operation and leaving behind abnormal parathyroid tissue, risking future recurrence. Cayo et al. analysed group of 755 patients who underwent parathyroidectomy and 163 (21.5 %) were found to have MGD on pathology. IOPTH monitoring was used in 161 of these cases. In 146/161 cases (90.7 %), the IOPTH level fell by at least 50 % after removal of all suspected abnormal glands. All of these patients (100 %) remained normocalcemic postoperatively. In 15/161 cases (9.3 %), the PTH level did not fall by >50 %. For 11/15 cases (73 %), patients remained hypercalcemic postoperatively or had recurrence. However, in the remaining four cases, the patients became normocalcemic postoperatively despite failure of the PTH to fall by >50 %. In each of these patients, PTH levels fell by 40–50 %. Thus, IOPTH monitoring accurately predicted success or failure of parathyroidectomy in 97.5 % (157/161) of patients with MGD. A fall of IOPTH by >50 % can be used as a highly accurate predictor of cure in patients with MGD [16].

McCoy et al. analysed initial parathyroid operations of 1150 patients treated in 1998–2012 and found that 15 % had MGD. MGD risk varied inversely with weight of first resected abnormal gland. Microadenoma required BNE more often than macroadenoma (48 vs. 18 %; *p* < 0.01). When at exploration the first resected gland was <200 mg, the rates of MGD (40 vs. 11 %; *p* = 0.001), inadequate initial IOPTH drop (67 vs. 79 %; *p* = 0.002), operative failure (6.6 vs. 0.7 %; *p* < 0.001), and long-term recurrence (1.6 vs. 0.3 %; *p* = 0.007) were higher. Thus, during exploration for sporadic pHPT, a first abnormal gland <200 mg should heighten suspicion of MGD and presages a tenfold higher failure rate [110].

The European Society of Endocrine Surgeons (ESES) recommended the use of IOPTH monitoring for patients undergoing ‘targeted’ parathyroidectomy on the basis of a single preoperative localization study. If preoperative localization with sestamibi and ultrasound is not concordant and the surgeon wishes to perform a minimally invasive ‘targeted procedure’, the use of intraoperative IOPTH monitoring is recommended. When preoperative localization with sestamibi and ultrasound is concordant for single-gland disease, the use of this adjunct is of little value. In addition, the use of IOPTH can be recommended in reoperative parathyroidectomy to lateralize hyperfunctioning parathyroid tissue (internal jugular vein/s sampling) when preoperative localization is uncertain or to predict cure and reduce the need for continued exploration in the scarred neck [103, 106].

### Is local anaesthesia with intravenous sedation feasible surgical treatment of sporadic MGD?

There are no reported cohorts addressing this issue.

## Long-term results of surgical treatment for sporadic multiglandular disease

### Does cure rate of surgery for sporadic MGD differ from outcomes of surgery for solitary parathyroid adenoma?/prevalence of persistent sporadic MGD

An accurate cure rate is impossible to calculate as one does not know exactly the value of the denominator (i.e. the exact number of patients with MGD in a population) and most estimates are based on how common is MGD demonstrated in patients with persistent pHPT after resection of a single adenoma.

Because patients with MGD are not expected to have concordant localization studies, they are more likely to be identified in the subgroup of patients with negative or non-concordant scans. For example, in a group of 492 patients, those with positive sestamibi scan results compared with those with negative results had a higher rate of single-gland disease (87 vs. 63 %, respectively) and lower rates of double adenoma (6 vs. 22 %, respectively) and asymmetric hyperplasia (7 vs. 15 %, respectively) (*p* < 0.001) [111]. Similarly, the incidence of MGD in a large series of 2185 patients was at least twice higher (12.8 vs. 5.4 %) in the subgroup of 836 (38 %) of patients with non-localising scans when compared with those with positive scans. The authors reported no difference in intraoperative success (93.9 vs. 95.6 %) or cure rates (96.2 vs. 97.7 %) between non-localised and localised groups [112].

Excellent cure rates were reported from Wisconsin University in a group of 161 patients with MGD identified within a cohort of 755 patients. In 146/161 cases (90.7 %), the IOPTH level fell by at least 50 % after removal of all suspected abnormal glands and all of these patients (100 %) remained normocalcemic postoperatively. In 15/161 cases (9.3 %), the PTH level did not fall by >50 % and 11 of these 15 patients remained hypercalcemic postoperatively or had recurrence. However, in the remaining four cases, the patients became normocalcemic postoperatively despite failure of the PTH to fall by >50 % [16].

Others have also reported that the failure to decrease IOPTH by >50 % and into normal range is more common in MGD than in patients with single adenomas (35.2 vs. 16.6 %) [113] and that a modified criteria of a drop in IOPTH of >75 % from baseline and within normal range should be used to predict adequate gland resection when MGD is identified during focused parathyroidectomy [15]. In their experience, the cure rate of MGD was less satisfactory as 14 of 193 (7.3 %) patients with pHPT had persistent disease [15].

The general trend is that large series report cure rates similar for MGD and for single adenomas. In a retrospective analysis of 1402 patients, the rate of persistent pHPT was higher among patients with double adenoma (4 %) vs. single adenoma (1.3 %) and hyperplasia (2.2 %; *p* = 0.0049) [10]. Wharry et al. analyzed 1108 initial parathyroid operations for sporadic primary hyperparathyroidism using IOPTH and reported that a long-term recurrence was more likely in patients with a final IOPTH level of 41–65 pg/mL than with a level ≤40 pg/mL (1.2 vs. 0 %; *p* = 0.016). Hence, patients with a final intraoperative iPTH level between 41 and 65 pg/mL should be followed up beyond 6 months for long-term recurrence [114].

The cure rate after excision of MGD could be influenced by the size of the remnant gland. This issue was addressed in a study of 172 patients who underwent subtotal parathyroidectomy (sPTX) for pHPT and were left with either a whole gland remnant (WGR; *n* = 108, 63 %) or a partial gland remnant (PGR; *n* = 64, 37 %) after sPTX. Cases with positive family history for pHPT were more likely to have a PGR (12.5 vs. 3.7 %; *p* = 0.03). Individuals with a PGR tended to have larger glands encountered by surgeons intraoperatively (525 ± 1308 vs. 280 ± 341 mg; *p* = 0.02). Overall, the cure rate was 97 % and recurrence rate of 5 % after a mean follow-up of 29 months [115].

### Prevalence of recurrent sporadic multiglandular disease

In a retrospective analysis of 1402 patients, the recurrence rate after a median follow-up time of 12 months was higher among patients with double adenomas (7.3 %) and MGD (4.4 %) vs. single adenomas (1.7 %; *p* = 0.0005). These data suggest that double adenoma in some cases could represent asymmetric or asynchronous hyperplasia. Hence, in situations where a double adenoma has been identified, the surgeon should have a high index of suspicion during surgery and postoperatively for the possibility of four-gland disease (grade C recommendation, GRADE: moderate, ⊕⊕⊕O) [11].

### Is more intense follow-up justified in sporadic multiglandular disease?

There is no data reported on this topic. A consensus statement should be issued based on ‘expert advice’.

### Redo operations in sporadic multiglandular disease

There are no reported cohorts addressing this issue.

## Summary

In the vast majority of patients with pHPT a single adenoma is the cause of disease. In literature, the percentage of sporadic MGD varies between 7 and 33 %. Of these, the majority is a hyperplasia involving all parathyroid glands whereas the remaining is double, or in very rare cases, triple adenomas.

Histopathology can (with a fair degree of accuracy) distinguish between a normal and pathological parathyroid gland, but be of limited aid to separate hyperplasia from adenoma. There are, however, some histopathological findings supporting parathyroid adenoma, fibrous capsule, solitary nodule, nuclear pleomorphism, normal rim, fewer fat cells and low/none intracellular fat. Hyperplasia on the other hand is supported by more than one gland (usually all four, but not mandatory) and involved nodule expansions of both chief and oxyphilic cells, no clear capsule, fewer fat cells, and absence of normal rim. However, needless to mention, there are significant overlap in pathological characteristics between adenoma and hyperplasia, and there are no convincing and useful histopathological criteria to differentiate between the two entities. At present and in the near future, the best clinical practice is likely a close cooperation between the pathologist and the surgeon. However, molecular biological methods may be a useful instrument in the future to distinguish and discriminate adenomas from hyperplasia.

Several issues demanded a comparison of published studies from different medical reports regarding preoperative diagnosis of sporadic MGD. As a consequence, it is difficult to make valuable statements on preoperative diagnosis of sporadic MGD with a sufficient recommendation rating. There is low evidence and recommendation from the literature for localization procedures for MGD.

Several limitation of the literature published to date have emerged from this review.

The results of localization procedures were not always compared with the results of neck exploration, definitive histology and postoperative calcium status at the first follow-up after discharge and were graded as follows: true preoperative localization of solitary adenoma (TP); false preoperative localization of solitary adenoma (FP); correct position of one pathological gland but MGD not predicted; false prediction of MGD in solitary adenoma; correct prediction of MGD; and lastly, negative/inconclusive preoperative examination [100]. Such a definition was not commonly applied to all investigations.

Only a small number of randomised trials comprising a comparatively modest number of patients have been published from specialised centres on localization procedures. A small number of studies analysed extensively preoperative diagnosis of sporadic MDG [100]. Moreover, localization procedures are not performed in all surgical procedures. In the survey by Bergenfelz et al., localization procedures were performed in 1831 of 2708 consecutively registered patients (68 %) sestamibi scintigraphy in 1473 patients (54 %), ultrasound in 1120 patients (41 %), CT in 62 patients (2 %), MRI in eight patients (0.3 %), venous sampling in 32 patients (1 %), and PET in 34 patients (1 %) [72]. Moreover, only in two trials (comprising 139 individuals), included patients that were not selected on the basis of the results of the preoperative localization procedures [70, 116].

Finally, the cost effectiveness for diagnostic procedures is debated for MGD. The argument regarding cost effectiveness is important but difficult to analyse given the variety in health care systems worldwide: waiting lists, costs for re-operation, sick leave, localization examinations, IOPTH etc. [103].

There is no level I or II evidence to inform which patients with pHPT should undergo BNE. Nevertheless, the presence of certain clinical risk factors for MGD should be taken into consideration in decision making which operative approach should be used for individual patient. BNE should be considered in MEN-1 syndrome, negative preoperative localization studies and inadequate decrease of IOPTH level following removal of the image-indexed parathyroid lesion. All other clinical scenarios can be regarded as relative indications for BNE and include isolated familial PHPT, MEN-2 syndrome, history of lithium therapy, head and neck irradiation in anamnesis or discordant preoperative localization studies [117–120].

There is conflicting and equally weighted low level evidence supporting a routine preoperative plan of bilateral neck exploration vs. selective unilateral exploration for LHA (no recommendation). The IOPTH assay can be utilised to confirm complete removal of all hyperfunctioning parathyroid tissue, which allows for termination of surgery with confidence that the hyperparathyroid state has been successfully corrected and to identify patients with additional hyperfunctioning parathyroid tissue following the incomplete removal of diseased parathyroid/s, which necessitates extended neck exploration in order to minimise the risk of operative failure. To achieve a high success rate of parathyroidectomy, the surgeon needs to be aware of intraoperative hormone dynamics during the case and carefully choose the protocol and interpretation criteria that best fit the individual practice. Understanding the nuances of IOPTH monitoring allows the surgeon for achieving intraoperative confidence in predicting operative success and preventing failure in cases of unsuspected MGD, while safely limiting neck exploration in the majority of patients with sporadic pHPT.

Conflicting data exist in the literature regarding the prevalence of persistent and recurrent disease following parathyroidectomy for sporadic MGD vs. single-gland disease. The general trend is that large series report cure rates similar for MGD and for single adenomas. However, some data suggest that double adenoma in some cases could represent asymmetric or asynchronous hyperplasia. Therefore, in situations where a double adenoma has been identified, the surgeon should have a high index of suspicion during surgery and postoperatively for the possibility of four-gland disease.

## Recommendations

- Level V evidence supports that most single adenomas are monoclonal lesions arising from a single precursor, whereas sporadic MGD is polyclonal, hence a single parathyroid adenoma and MGD represent two different diseases.
- Levels IV and V evidence supports that the majority of cases with double parathyroid adenomas are synchronous. This notion is based on the observation that few patients have a recurrent disease after a successful parathyroidectomy.
- Levels III to V evidence supports an etiologic link between sustained lithium therapy and both hypercalcemia and increased PTH serum level.
- Levels IV and V evidence do not support any differences with respect to gender, age and preoperative serum calcium and PTH levels between a solitary adenoma and sporadic MGD.
- Level V evidence do not support any differences in prevalence of a solitary parathyroid adenoma and sporadic MGD among patients with radiation-induced vs. non-radiation-associated pHPT.
- Levels III to V evidence supports that negative preoperative localization studies in pHPT are highly predictive of a small-sized solitary adenoma or MGD.
- Levels I to IV evidence supports that sestamibi scanning and US have an unsatisfactory accuracy in predicting sporadic MGD, which is significantly inferior to a solitary parathyroid adenoma.
- Level V evidence supports that 4D-CT identifies more than a half of abnormal parathyroids in MGD missed by traditional imaging. However, taking into consideration that 4D-CT carries a huge radiation exposure, BNE remains the gold standard surgical approach having a high success rate in experienced hands in cases with negative or discordant sestamibi and ultrasound (grade C recommendation, GRADE: high, ‘⊕⊕⊕⊕’).
- Level V evidence supports that CT-MIBI-SPECT image fusion is superior to CT or MIBI-SPECT alone in preoperative localization of all pathologic glands in patients suffering from MGD (grade C recommendation, GRADE: high, ‘⊕⊕⊕⊕’).
- Levels III and IV evidence supports that genetic testing should be undertaken in patients with seemingly sporadic MGD under 40 years of age to rule out hereditary parathyroid disease (grade C recommendation, GRADE: high, ‘⊕⊕⊕⊕’).
- Levels III to V evidence supports that the presence of certain clinical risk factors for MGD should be taken into consideration in decision making which operative approach should be used for individual patient. Hence, BNE should beconsidered in MEN-1 syndrome, negative preoperative localization studies, and inadequate decrease of IOPTH level following removal of the image-indexed parathyroid lesion. All other clinical scenarios can be regarded as relative indications for BNE and include isolated familial PHPT, MEN-2 syndrome, history of lithium therapy, head and neck irradiation in anamnesis or discordant preoperative localization studies (grade C recommendation, GRADE: moderate, ⊕⊕⊕O).
- Levels IV and V evidence supports the use of preoperative parathyroid imaging if a unilateral/focused exploration is planned in case of suspicion of sporadic MGD (grade C recommendation, GRADE: moderate, ⊕⊕⊕O).
- Levels IV and V evidence supports the use of IOPTH monitoring to guide appropriate surgical therapy in sporadic MGD (grade C recommendation, GRADE: moderate, ⊕⊕⊕O).
- There is conflicting and equally weighted level V evidence supporting a routine preoperative plan of BNE vs. unilateral neck exploration for selected patients with MGD, e.g. LAH (no recommendation).
- There is conflicting or equally weighted levels IV to V evidence supporting that cure rates can be similar or worse for sporadic MGD than for single adenomas. Best outcomes can be expected if surgery is performed by an experienced parathyroid surgeon working in a high-volume parathyroid surgery centre (grade C recommendation, GRADE: high, ‘⊕⊕⊕⊕’).
- Levels IV and V evidence supports that recurrent and persistence pHPT occurs more frequently in patients with double adenomas. Hence, in situations where a double adenoma has been identified, the surgeon should have a high index of suspicion during surgery and postoperatively for the possibility of four-gland disease (grade C recommendation, GRADE: moderate, ⊕⊕⊕O).
